# Social and behaviour change communication to improve child feeding practices in Ethiopia

**DOI:** 10.1111/mcn.13231

**Published:** 2021-06-15

**Authors:** Abdulhalik Workicho, Sibhatu Biadgilign, Meghan Kershaw, Rahel Gizaw, Jennifer Stickland, Wossen Assefa, Cherinet Abuye, Behailu Woldegiorgis, Lioul Berhanu, Eileen Kennedy

**Affiliations:** ^1^ Friedman School of Nutrition Science and Policy Tufts University Boston Massachusetts USA; ^2^ Growth through Nutrition Save the Children International Addis Ababa Ethiopia

**Keywords:** behaviour, child nutrition, complementary feeding, infant and child nutrition, infant feeding behaviour, maternal nutrition

## Abstract

Social and behaviour change communication (SBCC) interventions can positively affect optimal nutritional practices. This study evaluated the added value of a virtual facilitator tool to an enhanced community conversation (ECC) programme to improve infant and young child feeding (IYCF) practice among children under the Growth through Nutrition Activity programme in Ethiopia. The study used a quasi‐experimental design with a control group. Pregnant and/or lactating women were the study population for both study groups. The intervention (ECC + VF) group received all the same components as the control group but had the addition of in‐person ECC meetings supplemented with audio‐recorded virtual facilitators (VF) sessions designed to complement the monthly meeting lesson or topic. A difference in difference analysis was employed using generalized linear mixed model (GLMM) in Stata version 15.0 (Stata Corporation, College Station, TX). A *p*‐value of less than or equal to 0.05 was considered significant for all tests. Accordingly, a 13.6% change in iron folic acid (IFA) intake for 3 months and above was observed in the intervention group. Even though not statistically significant, large to moderate positive changes in child minimum diet diversity (20%), minimum acceptable diet (18%) and women diet diversity (7.9%) were observed in the intervention group. This study identified the use of virtual facilitators as a modality to transmit standard nutrition messages during ECC programmes for optimal IYCF practices. The findings strengthen the notion that using a combination of SBCC approaches has advantage over a single method in improving important nutritional practices.

**Key Messages**

- Using multiple communication strategies increases the positive effect of SBCC on child feeding practices.
- Proportion of women who exclusively breastfed increased from what has been noted in the baseline.
- Child minimum dietary diversity, minimum acceptable diet and women dietary diversity have improved by 20%, 18% and 7.9% from their baseline values, respectively.
- There were improvements in women's dietary diversity from baseline to follow up and IFA intake for three months and above.
- Tailor the use of virtual facilitators to focus on indicators that are most likely to be positively affected.

## 1 INTRODUCTION

The scope of undernutrition among children in low‐ and middle‐income countries (LMICs) spanned from acute to chronic malnutrition and several micronutrient deficiencies (Black et al., 2013; Tzioumis & Adair, 2014). These nutritional insults often translate into impaired cognitive and physical development for children and the perpetuation of the intergenerational cycle of malnutrition (Galler & Rabinowitz, 2014; Mericq et al., 2017; Tzioumis & Adair, 2014). Among various nutrition sensitive and specific interventions, social and behaviour change communication (SBCC) can play a pivotal role in promoting optimal nutritional practices among target populations (Hoddinott et al., 2018; Kennedy et al., 2018; Kim et al., 2016; Ruel & Alderman, 2013). Applications of SBCC have been demonstrated in nutrition programming and incorporated into agriculture, social safety net, early child development and school health programmes (Hoddinott et al., 2018; Lamstein, Koniz‐Booher, et al., 2014; Rennie et al., 2014; THEMANOFFGROUP, 2019). Moreover, various studies, both in Ethiopia and elsewhere, indicated that the use of multiple platforms in delivering SBCC interventions was found to significantly improve child feeding practices and nutritional status (Purnima Menon et al., 2020). A package of intensified SBCC interventions through interpersonal communications (IPC), agricultural activities, community mobilization (CM) and mass media (MM) resulted in significant improvements in minimum dietary diversity, minimum acceptable diet and stunting among children in Ethiopia (Kim et al., 2019). In a similar study in Bangladesh and Vietnam, strategies combining IPC, CM and MM to deliver SBCC interventions were found to be more effective in improving breastfeeding practices compared with a less intensive standard counselling method (P. Menon et al., 2016).

The Feed the Future Ethiopia, Growth through Nutrition Activity was designed to improve the nutritional status of women and young children in Ethiopia's four agricultural productive regions of Tigray, Southern Nations, Nationalities, and People (SNNP), Amhara, and Oromia, by focusing on the first 1000 days (from conception to age two). Growth through Nutrition used a comprehensive, intensive, multi‐sectoral nutrition programming at community levels, linking development and emergency efforts to build resiliency and sustainability of the nutritional and livelihood aspects of the programme. The project mainly focused on 110 targeted district/woredas across the four regions with the aim of addressing stunting reduction through health service improvements, increased production and availability of nutritious food, WASH products and services, as well as SBCC for improved health, nutrition, food security, livelihood and WASH behaviours (SavetheChildrenInternational, 2017).

Improving key positive practices requires change at the individual, household and community levels and requires services targeting both mothers and whole families. Although SBCC is a recognized tool for the delivery of high impact, cost‐effective nutrition interventions, there is still limited research on the effectiveness of SBCC strategies including their specific contribution to achieving optimal infant and young child feeding practices (Gillespie et al., 2013; Lamstein, Stillman, et al., 2014). Building on recent global evidence that SBCC theories and approaches are effective for improving nutrition outcomes, many programmes include SBCC as part of their main strategy to improve maternal and child nutritional status (Kim et al., 2016; Rennie et al., 2014; THEMANOFFGROUP, 2019). Within its large‐scale multisectoral project, the SBCC messages from Growth through Nutrition are distributed via communication materials (print and other media) and enhanced community conversations (ECCs), which are regular in‐person group meetings facilitated by a trained personnel that promote the adoption of nutrition‐related skills, behaviours and gender transformative roles, help people make positive changes within the contexts of their household and family environments, and use interactive methods and activities to help adults learn and adopt new behaviours. The project provided consistent, locally adapted, actionable messages which were reinforced at each project level in hopes of significant improvements in the short term and sustainable progress in the long term (SavetheChildrenInternational, 2017). In addition to the existing SBCC approach, a new tool designed to complement the ECCs, a pre‐recorded audio message with actors modelling the desired knowledge and behaviours called virtual facilitator (VF). This tool was anticipated to make the SBCC programme more robust and scalable. VFs, as one of first 1000 days SBCC materials, were an important component of the multimedia packages, which were designed by the project for use during ECC programmes. The materials facilitate the peer group activities, which consist of listening to nutrition‐related information, discussing nutrition‐related information and experiences, singing nutrition songs, demonstrations, skills‐building games and role play (THEMANOFFGROUP, 2019). The present research contributes to the body of evidence by investigating the benefits of a new SBCC tool (VF) that has been added to the existing ECC approach to enhance effects of SBCC within the Growth through Nutrition.

## 2 METHODS

### 2.1 Study setting and design

The study was conducted in three woredas (districts) of the Amhara and Oromia regions (Basoliben, Becho and Girar Jarso) of Ethiopia where Growth through Nutrition operates. The regions and districts were selected purposely and represent the various agroecological zones within Ethiopia. Within each district, two kebeles, one intervention and one control were selected to be included in the study. The study used a quasi‐experimental design. Quantitative data on outcome measures were collected at baseline and follow up from an intervention group (ECC + VF) and a control group (ECC only). The study lasted for 10 months. Data were collected from the same households in both rounds. The baseline data collection was conducted in December 2018, whereas the follow up data collection was completed in November 2019.

### 2.2 Intervention

The VF activities consisted of listening and discussing nutrition‐related information and experiences such as singing nutrition songs, demonstrations, skills‐building game and role play. The materials, combined with the on‐going comprehensive enhanced community conversations (ECC) activities, supported an *experiential learning approach* to help mothers, fathers and grandmothers build on the knowledge, attitude, experiences and skills they already have and, in turn, practice the adoption of positive behaviours that also encompass transformative gender roles. The intervention (ECC + VF) group received the ECC components supplemented with audio‐recorded VF sessions designed to complement the monthly meeting lesson or topic. The VF tool was pre‐recorded audio material using the voices of professional actors who played the roles of two fictional characters, *Ete Birtukan* and *Aya Mulat*. *Ete Birtukan* is an older woman who is a nurse and an expert in maternal and child nutrition. *Aya Mulat* is her husband and a retired agriculture extension worker who is knowledgeable on nutrition and nutrition‐sensitive agriculture. The VF audio recordings are played on a mobile phone or audio player and are designed to be turned off by the community change agent (CCA) when he or she hears a bell that signals the time to stop listening and start the group activity. The messaging included but was not limited to maternal and child health service utilization, antenatal care, nutrition counselling, initiation of breastfeeding, exclusive breastfeeding, complementary feeding, dietary diversity, role of husbands in childcare and nutrition, women empowerment in household decision making, and nutrition communication. The messaging was rolled out in 10 modules, each module taking 1 month, for a total of 10 months. This approach ensured transmission of similar messages for all participants and created a common understanding of the materials provided.

In the control group, the participants received monthly ECC meetings led by CCAs that facilitate the interaction among the group of 10–15 participants. In addition to this, the ECC provides take‐home materials with behaviours or activities to discuss and try at home level within the family and monitored and supervised during home visits scheduled by CCAs.

The Growth through Nutrition SBCC managers in the study areas monitored the progress of the activity done by CCAs who were trained in implementing ECC sessions. At initial stages of the project implementation, the managers had a training on project monitoring and the overall objectives of the study. Quality control methods were put in place to ensure that each participating group received the activity in the exact same way in terms of the time of the session, frequency, duration, order and content in each district and kebele.

In each woreda, two kebeles (for a total of six kebeles), were selected for this research purpose by the SBCC team, one kebele to receive the intervention and other kebele to be a control. Participants were pregnant and/or lactating women who were Growth through Nutrition beneficiaries in the selected livelihood programme. They were pre‐selected from a beneficiary list based on their enrolment in the ECC groups. The participants' assignment was evenly distributed across the selected kebeles, with each participant assigned to an intervention group based on the assignment of their kebele of residence.

### 2.3 Measurement

The study team of experienced data collectors collected the data before the start of the ECC programme in the study area (baseline) and then again after the completion of the programme approximately 10 months later. The questionnaire was designed to include demographic characteristics and other maternal and child health and nutrition indicators, targeted by the ECC and ECC + VF interventions. In this research, we have used the WHO guideline to define the maternal and child nutrition indicators; Minimum Dietary Diversity (MDD) was defined as the proportion of children 6–23 months age who received food from four or more groups. Minimum Meal Frequency (MMF) was defined as the proportion of breastfed and non‐breastfed children 6–23 months of age who received solid, semi‐solid or soft foods (including milk for non‐breastfed children) the minimum number of times. As outlined in the guideline, the minimum number would be two times for breastfed infants 6–8 months, three times for breast‐fed infants 9–23 months and four times for non‐breastfed children 6–23 months. Minimum Acceptable Diet (MAD) was defined as proportion of children 6–23 months of age who received a minimum acceptable diet apart from breast milk (WHO, 2010). All the data are based on maternal or care givers reporting. The women's diet diversity was based on a 24‐h qualitative recall. Accordingly, women who consumed five or more food groups from a 10‐food group category were defined to have a minimally adequate diet (FAO & FHI360, 2016).

### 2.4 Sample size estimations

The sample size was determined using the difference between two proportions of the intervention groups and control group. The sample was calculated using Gpower 3.1.9 with the following parameters: from recent survey findings, the proportion of children aged 6 to 23 months (both breastfed and non‐breastfed) who met minimum diet diversity (MDD) was 16.1% (82 out of the 508). This proportion was identified from the control group in the Baseline Study for Feed the Future Ethiopia Growth Through Nutrition Activity (SavetheChildrenInternational, 2017). By hypothesizing that the prevalence of minimum diet diversity would be improved by 15% in this study, proportion in intervention group was 31.1% (15% + 16.1%). The level of confidence was taken to be 95% with study power set at 80%. Then the sample size was 250. By considering 10% contingency for loss to follow‐up and by adding 1.5 design effect for the sampling variations and clustering, the total sample size was calculated to be 414, divided evenly between the intervention and control groups.

### 2.5 Statistical analysis

Quantitative data were recorded using Open Data Kit (ODK) questionnaires on Wi‐Fi‐enabled handheld electronic tablets. The data were exported for cleaning, editing and analysis using statistical package Stata version 15.0 (Stata Corporation, College Station, TX). Simple frequencies were generated to see the overall distribution of the variables of interest between the two groups and between the baseline and follow up. Significant differences within the groups was measured using *χ* ^2^ test. To examine changes between groups (intervention vs control) at baseline and follow‐up assessments, a difference in difference analysis was performed using generalized linear mixed model (GLMM) assuming a normal distribution and taking the clustering effect at kebele level into account. The coefficients of all the indicators were determined from the interaction between the grouping variable (control or intervention group) and time (baseline or follow up). For ease of interpretation, the coefficients and their respective confidence intervals were changed into percentages. These represent the per cent of change from the baseline to follow up reflecting an intervention effect. All continuous data were checked for normality. All tests performed were two sided, and a *p*‐value of less than or equal to 0.05 was considered significant for all tests. The study was approved by the Tufts University Institutional Review Board. All study participants were informed about the purpose of the study, and their consent was obtained before being interviewed.

## 3 RESULTS

### 3.1 Socio‐demographic characteristics

A total of 410 participants were ultimately enrolled in the study during the baseline. There was a 5% loss to follow up during the follow up round due primarily to relocation and refusal to participate. A total of 390 repeat participants were included in the follow up survey, which is the basis of the analysis to identify the intervention effect. Participants were evenly split across regions, woredas and intervention group.

The socio‐demographic characteristics of the participants in intervention and control groups we similar. A small minority of households were female‐headed (<7%). Most of the participants in both groups were 29 years old or younger and had not received any schooling (Table 1). For those women who had any education, primary school was the largest category. Like females, most males had no formal education. Most of both groups had a household size of five persons or greater. Many respondents were married. At baseline, the largest group of children was less than 6 months and at follow‐up 12 to 15 months.

**TABLE 1:** Socio‐demographic characteristics of participants by survey round

Variable		Baseline *n* (%)		Follow‐up *n* (%)	
		Control	Intervention	Control	Intervention
Region	East Oromia	67 (33)	69 (34)	64 (32)	63 (33)
	West Oromia	69 (34)	67 (33)	67 (34)	64 (33)
	Amhara	69 (34)	69 (34)	67 (34)	65 (34)
Woreda	Basoliben	69 (34)	69 (34)	67 (34)	65 (34)
	Becho	69 (34)	67 (33)	67 (34)	63 (33)
	Girar Jarso	67 (33)	69 (34)	64 (32)	64 (33)
	Only adult women	13 (6)	6 (3)	11 (6)	5 (3)
Household size	≤4 members	91 (44)	91 (44)	78 (39)	72 (38)
	≥5 members	114 (56)	114 (56)	120 (61)	120 (63)
Age of the participants	≤29	120 (60)	114 (56)	114 (59)	106 (56)
	30–34	47 (24)	42 (21)	37 (19)	44 (23)
	35–39	26 (13)	37 (18)	33 (17)	33 (17)
	>39	6 (3)	10 (5)	11 (6)	8 (4)
Age of youngest child	<6 month	44 (31)	51 (36)	11 (6)	12 (7)
	6–8 months	33 (23)	41 (29)	31 (16)	22 (13)
	9–11 months	32 (23)	24 (17)	40 (21)	35 (20)
	12–15 months	25 (18)	21 (15)	64 (33)	62 (36)
	16–23 months	8 (6)	4 (3)	47 (24)	41 (24)
Mother any schooling	Yes	73 (36)	84 (41)	70 (35)	73 (38)
	No	132 (64)	121 (59)	128 (65)	119 (62)
Adult female education level	No education	1 (1)	2 (2)	1 (1)	0 (0.00)
	Primary (1–8 grade)	64 (88)	62 (78)	61 (87)	56 (77)
	Secondary (9–12 grade)	8 (11)	17 (20)	8 (12)	14 (19)
	College and above	0 (0.00)	3 (4)	0 (0)	3 (4)
Household adult male education level	No education	115 (62)	93 (48)	109 (55)	85 (44)
	Primary	56 (30)	77 (40)	60 (30)	72 (38)
	Secondary	14 (8)	15 (8)	17 (9)	16 (8)
	College and above	0 (0)	7 (4)	0 (0)	4 (2)
Marital status	Never married	3 (2)	3 (2)	2 (1)	4 (2)
	Married	193 (94.15)	194 (95)	187 (94)	184 (96)
	Others	9 (4)	8 (4)	11 (5)	4 (2)

### 3.2 Child diet and nutrition

Table 2 shows that there was little change between baseline and follow up in the control group in the percentage of breast milk that was discarded; in the intervention group, however, the percentage of women in the intervention group who discarded breast milk increased from 45.4% to 56.4% from baseline to follow‐up.

**TABLE 2:** Infant and young child feeding (IYCF) practices

Variable		Baseline		*p*‐value	Follow up		*p*‐value
		Control, *n* (%)	Intervention, *n* (%)		Control, *n* (%)	Intervention, *n* (%)	
Throw away the first milk	Yes	78 (55)	64 (45)	0.095	103 (53)	97 (56)	0.474
	No	63 (45)	77 (55)		90 (47)	74 (43)	
Initiation of breastfeeding	Immediately	96 (69)	98 (70.00)	0.168	129 (67)	129 (75)	0.118
	1 to 24 h	37 (27)	29 (21)		51 (27)	38 (22)	
	24+ h	6 (4)	13 (9)		11 (6)	4 (2)	
Taken other than breast milk in 6 months	Yes	86 (60)	82 (58)	0.503	81 (42)	68 (40)	0.130
	No	56 (40)	59 (42)		111 (58)	103 (60)	
Timely initiation of comp. Food	Yes	77 (80)	72 (95)	0.065	169 (93)	154 (96)	0.042*
	No	9 (11)	4 (5)		13 (12)	4 (3)	
Minimum dietary diversity	Low	88 (90)	79 (88)	0.661	125 (69)	100 (63)	0.243
	Appropriate	10 (10)	11 (12)		57 (31)	60 (38)	
Minimum meal frequency (MMF)	Yes	47 (48)	39 (43)	0.525	148 (88)	131 (84)	0.288
	No	51 (52)	51 (57)		20 (12)	25 (16)	
Minimum acceptable diet (MAD)	Yes	6 (6)	5 (6)	0.869	53 (27)	51 (27)	0.529
	No	92 (94)	85 (94)		129 (66)	107 (56)	
Bottle feeding	Yes	15 (17)	8 (11)	0.219	21 (12)	32 (20)	0.027*
	No	72 (83)	68 (89)		161 (89)	128 (80)	

* *p*‐value significant at 0.05 level.

All the children in the study reported to be breastfed in both groups. Similarly, in the follow up study, 67.2% of infants in the control group and three‐quarters (75.4%) from the intervention group were reported to have been breastfed immediately after birth, with no statistically significant difference in the practice. The baseline data also showed no difference across the two groups in the initiation of breastfeeding. Though there was an approximately 18% decrease from the baseline to follow up in providing any food other than breast milk to the child prior to 6 months in the intervention groups, the difference between the control and intervention groups during both baseline and follow‐up studies was not statistically significant. The average age when the mothers gave their child something other than breast milk (including water) for both groups was 5 months. The difference across the two groups in terms of feeding a child any food other than breast milk was not significant as a similar proportion; 93.8% of children from the control and 93.6% from the intervention group only gave the child breast milk.

The study findings indicated that approximately 32% and 38% of children in the control and intervention groups, respectively, at the end of the study, had an appropriate minimum dietary diversity. Comparably, approximately 27% of the children from both groups had a minimum acceptable diet at time of follow‐up. Though there was improvement from the baseline values, the difference in the proportion between the control and intervention groups both during the baseline and follow‐up studies was not statistically significant. A statistically significant difference was identified in terms of bottle‐feeding and feeding of solid or semi‐solid foods among children in the control and intervention groups during the follow‐up study. Surprisingly, the per cent of women bottle feeding increased from 10.5% (baseline) to 20% at the end point. There was a significant difference between the control and intervention groups in the number of meals the mothers reported eating while pregnant, with over 50% of both groups reporting eating ‘more’ while they were pregnant and breastfeeding. Maternal dietary diversity increased between the two time periods for both intervention and control groups. The differences between the control and intervention women consuming diversified diets were not statistically significant.

**FIGURE 1:**
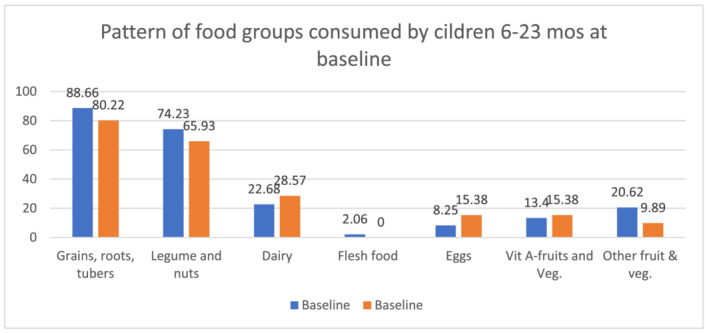
Pattern of food groups consumed by children 6–23 months during the baseline survey

**FIGURE 2:**
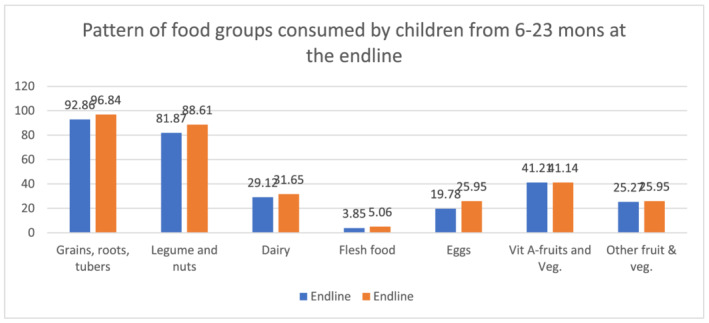
Pattern of food groups consumed by children 6–23 months during the follow up survey

The pattern of consumption of different foods was also assessed among children from 6 to 23 months of age. At baseline, majority of the children from the two groups were fed diets which included grains, roots, tubers, legumes and nuts (Figure 1). This pattern remained mostly the same during the follow up period but with significant improvements in the consumption of legumes and nuts, dairy, eggs and vitamin A‐rich fruits and vegetables (Figure 2). The difference in the consumption of legumes and nuts both in the control group (*p* = 0.04) and in the intervention group (*p* = 0.02) between the two time points was statistically significant. A statistically significant difference was also identified both in the control (*p* = 0.03) and intervention (*p* = 0.02) groups from baseline to follow up in the consumption of vitamin A‐rich fruits and vegetables. The study also identified an improvement in the consumption of eggs and dairy products from baseline to follow up. The change was statistically significant in the consumption of eggs both among control (*p* = 0.03) and intervention (*p* = 0.04) groups. Though there was an improvement in diary consumption among the intervention group from baseline to follow‐up, the change was significant only among the control group (*p* = 0.04).

### 3.3 Results of the difference in difference analysis

The intervention has brought about large improvements in dietary patterns, even though the changes were not statistically significant. The child minimum dietary diversity, minimum acceptable diet and women dietary diversity have improved by 20%, 18% and 7.9% from their baseline values, respectively (Tables 3 and 4).

**TABLE 3:** Maternal dietary practices

Variable		Baseline		*p*‐value	Follow up		*p*‐value
		Control, *n* (%)	Intervention, *n* (%)		Control, *n* (%)	Intervention, *n* (%)	
While breastfeeding you eat	More	35 (41)	32 (43)	0.429	102 (56)	93 (58)	0.030*
	Same	40 (47)	38 (51)		61 (34)	62 (39)	
	Less	11 (13)	5 (7)		19 (11)	5 (3)	
Minimum dietary diversity for women (MDD‐W)	Low (<5)	158 (77)	160 (78)	0.813	136 (69)	118 (62)	0.151
	Appropriate (≥5)	47 (23)	45 (22)		61 (31)	72 (38)	

* *p*‐value significant at 0.05 level.

**TABLE 4:** Estimates from difference in difference analysis

Outcome	β coefficient	β coefficient (%)	Robust Stand. Error	95% CI	*p*‐value
Minimum dietary diversity (appropriate)	0.20	20%	0.17	(−14%, 54%)	0.248
Minimum meal frequency (yes)	0.02	2%	0.14	(−27%, 30%)	0.912
Minimum acceptable diet (yes)	0.18	18%	0.17	(−16%, 53%)	0.295
Women dietary diversity	0.08	8%	0.06	(−4.5%, 20.3%)	0.213
Antenatal (ANC) follow‐up (yes)	0.013	1.3%	0.04	(−11%, 9%)	0.812
Iron and folic acid (IFA) intake (yes)	0.034	3.4%	0.05	(−7%, 14%)	0.545
Duration of IFA intake (3 months and more)	0.14	14%	0.08	(−22%, 49%)	0.045*

* *p*‐value significant at 0.05 level.

## 4 DISCUSSION

The study evaluated the added effects of the VF added to the existing ECC programme for improving the diets of children. Positive changes were noted in infant and young child feeding practices. There were improvements in a child's minimum dietary diversity, minimum acceptable diet, women's dietary diversity from baseline to follow up and IFA intake for 3 months and above. These study findings corroborate prior reviews of effective infant feeding programmes, which highlight the importance of using a variety of SBCC interventions to encourage changes in individual behaviours and social norms (Lamstein, Stillman, et al., 2014). For example, a study to evaluate the effect of exposure to large scale SBCC interventions in two regions in Ethiopia (SNNP and Tigray) showed that there were large improvements in terms of infant and young child feeding practices that were attributed to their exposure to SBCC interventions (Kim et al., 2016).

Findings from other studies suggest that increasing the frequency of exposure and using multiple communication strategies increase the positive effect of SBCC on maternal and child feeding practices (Kim et al., 2016; Lamstein, Stillman, et al., 2014). Although the body of literature on the effectiveness of SBCC to improve women's dietary practices during pregnancy and lactation is small, the existing evidence, however, indicates that SBCC approaches can and do succeed in improving uptake of behaviours to diversify diet (Pelto et al., 2016). The findings in the present study reinforce the evidence of the effect of SBCC on complementary feeding practices. The major limitation in the current published literature on SBCC is the diversity of indicators used to measure complementary feeding, making a comparison across studies a challenge.

A randomized controlled trial in Bangladesh indicated that SBCC interventions resulted in a sustained positive effect on infant and young child feeding knowledge among mothers over time. This same study also reported that the SBCC effects on improved knowledge of infant and young child diet and nutrition persisted 6–10 months after the intervention ended. Much of the gain in knowledge was achieved in the first 12 months of the intervention (Hoddinott et al., 2018). Other studies indicated a strong correlation between the number of communication channels used during SBCC intervention and increased knowledge; it is worth noting that printed media such as stickers, posters and leaflets were associated with significantly higher knowledge scores than were other channels (Lamstein, Stillman, et al., 2014). Where a strong sense of community spirit existed, friends relatives and neighbours were more likely to adopt positive changes and practices. Therefore, for a favourable impact on IYCF practices, SBCC interventions need to be implemented through communities and households using a multisectorial approach supported by actions from a range of stakeholders.

Findings from the analysis in the current study indicated that there were improvements in both the control and intervention groups between baseline and follow up. To this end, the results document improvements in child diet diversity and minimum acceptable diet both in control and intervention groups between the two time periods. Other studies also indicated that different types of SBCC approaches increased the consumption of diversified diet among children. The combination of audio‐visual media and individual counselling and private sector advertisements was effective to make mothers aware of optimal complementary feeding practices and brought about a significant change in the practice within a short time period (Bhandari et al., 2004; Kilaru et al., 2005; Lamstein, Stillman, et al., 2014; Roy et al., 2007; Sun et al., 2011; Zaman et al., 2008).

The proportion of women who exclusively breastfed increased from what has been noted in the baseline, even though there still are significant number feeding their infants things other than breast milk in the first 6 months. Child minimum dietary diversity, minimum acceptable diet and women dietary diversity have improved by 20%, 18% and 7.9% from their baseline values respectively. Community‐based SBCC interventions with various approaches, intensity and type were found to significantly improve the practice of exclusive breastfeeding. Evidence from systematic reviews and primary studies revealed that the rate and duration of breastfeeding were positively affected by interventions. The studies also observed a relatively greater effect of these community‐based breastfeeding promotion interventions among participants in developing countries (Hall, 2011; Imdad et al., 2011; Jolly et al., 2012). Similarly, there was a positive change in the consumption of legumes and nuts, dairy, eggs and vitamin A‐rich fruits and vegetables from the baseline value. After 6 months of the infant's life, breast milk alone no longer meets all the nutritional need of the growing infant. This leads to timely initiation of optimal complementary feeding, which is critical to the infant's health development and growth. According to studies, the adoption of optimal complementary feeding practices and other supportive strategies could avert a 17% stunting prevalence by the age of 24 months (ref lancet). SBCC strategies are found to be effective in promoting optimal IYCF practices hence are embedded in various nutrition sensitive interventions. Studies have also documented that various forms of SBCC interventions positively affected the consumption of animal source foods, fruits, vegetables, legumes and egg (Lamstein, Stillman, et al., 2014; Palwala et al., 2009; Penny et al., 2005; Vazir et al., 2013). A statistically significant change from the baseline, which can be attributed to the intervention, was also exhibited in terms of IFA intake for 3 months and above.

In conclusion, large to moderate positive changes from baseline were observed among participants in the intervention group for most of the outcome indicators. First, it is important to strengthen the use of VFs as a modality to transmit nutrition messages during the ECC programmes for a positive change in IYCF behaviours. Second, as the findings showed a varying magnitude of changes for the outcomes studied, it is equally important to tailor the use of VFs to focus on indicators that are most likely to be positively affected. Finally, a continuous monitoring and evaluation of the ECC implementation are of paramount importance to ensure fidelity across the intervention and to harness its maximum benefit. This study used a quasi‐experimental design with a control group adequately powered to answer the research questions. The baseline and follow up surveys were conducted during the same season 10 months apart to control any seasonality effect. Nevertheless, the study has some limitations. Inherent to the study design used, it would be difficult to control the effects of confounding variables that may arise from the lack of randomization and mask the actual effects of the intervention in bringing about the desired changes. Additionally, due to the time laps between the two surveys, there was a change in sample characteristics, which might have affected the observed changes between the two time points. Even though large changes were reported, some of the estimates for IYCF indicators are found to be less precise as their wide confidence intervals indicated. This is mainly due to the sample differences during baseline and follow up measurements, as children who were not eligible (<6 months old) for assessment of the indicators during the baseline were old enough and measured for the specific indicators during the follow up study. A study with a larger sample size, stronger design and more kebeles under the intervention and control groups would be needed to identify the real effects of the intervention. Therefore, it is important to consider these limitations while interpreting the findings.

## CONFLICTS OF INTEREST

The authors declare that they have no conflicts of interest.

## CONTRIBUTIONS

AW and SB conceived and designed the protocol, analysed the data, interpreted the results and wrote the manuscript. MK, RG, JS, EK, CA, WA, BW and LB assisted in data analyses, the interpretation of the results and reviewing the manuscript. All authors have seen and approved the final version of the manuscript.

## Data Availability

The data that support the findings of this study are available from the corresponding author upon reasonable request.
